# In Vitro Evaluation of Antioxidant, Anti-Inflammatory, Antimicrobial and Wound Healing Potential of *Thymus Sipyleus* Boiss. Subsp. *Rosulans* (Borbas) *Jalas*

**DOI:** 10.3390/molecules24183353

**Published:** 2019-09-14

**Authors:** Oya Ustuner, Ceren Anlas, Tulay Bakirel, Fulya Ustun-Alkan, Belgi Diren Sigirci, Seyyal Ak, Huseyin Askin Akpulat, Ceylan Donmez, Ufuk Koca-Caliskan

**Affiliations:** 1 Department of Pharmacology and Toxicology, Faculty of Veterinary Medicine, Istanbul University-Cerrahpasa, Avcilar, 34320 Istanbul, Turkey; oyakeles@istanbul.edu.tr (O.U.); tulbakir@istanbul.edu.tr (T.B.); fustun@istanbul.edu.tr (F.U.-A.); 2 Department of Microbiology, Faculty of Veterinary Medicine, Istanbul University-Cerrahpasa, Avcilar, 34320 Istanbul, Turkey; belgis@istanbul.edu.tr (B.D.S.); sak@istanbul.edu.tr (S.A.); 3 Department of Biology, Faculty of Education, Sivas Cumhuriyet University, 58140 Sivas, Turkey; aakpulat99@yahoo.com; 4 Department of Pharmacognosy, Faculty of Pharmacy, Gazi University, 06330 Yenimahalle-Ankara, Turkey; ceylanaka@gazi.edu.tr (C.D.); ukoca@gazi.edu.tr (U.K.-C.)

**Keywords:** *Thymus sipyleus* Boiss. subsp. *rosulans* (Borbas) *Jalas*, wound healing, antioxidant, anti-inflammatory, antibacterial

## Abstract

*Thymus sipyleus* Boiss. subsp. *rosulans* (Borbas) *Jalas* (TS) is a commonly used plant in the treatment of various complaints, including skin wounds in Turkish folk medicine. Despite the widespread traditional use of TS, there is not any scientific report confirming the effectiveness of this plant on the healing process. This research aimed to investigate the effects of different extracts obtained from TS on biological events during wound healing, on a cellular basis. In this context, proliferative activities of the extracts, as well as the effects on wound closure and hydroxyproline synthesis, were determined. In addition to wound healing properties, the antioxidant, antibacterial and anti-inflammatory activities of the extracts were evaluated. Decoction (D) and infusion (I) extracts contained the highest amount of phenolic content and showed the most potent activity against DPPH radical. All extracts exhibited complete protection against the damage induced by hydrogen peroxide (H_2_O_2_) by increasing cell viability compared to only H_2_O_2_-treated groups, both in co-treatment and pre-treatment protocols. None of the extracts exhibited cytotoxic activity, and most of the extracts from the TS stimulated fibroblast proliferation and migration. All TS extracts exert anti-inflammatory activity by suppressing the overproduction of tumor necrosis factor-alpha (TNF-α) and nitric oxide (NO). The most pronounced activity on hydroxyproline synthesis was observed in D extract. In summary, it was observed that TS extracts can promote the healing process by enhancing fibroblast migration, proliferation and collagen synthesis as well as suppressing pro-inflammatory cytokines. The obtained data in this work support the traditional use of TS as a valuable plant-based compound for the treatment of wounds.

## 1. Introduction

The acute and chronic wound problem is a growing global concern for the resource-poor and developed world. In a retrospective analysis of Medicare beneficiaries in 2018, it has been reported that 8.2 million people were found to have infectious or non-infectious wounds and the cost of treatment for wounds ranged from $ 28.1 billion to $96.8 billion. The increasing costs for health care, as well as the threat of infections that are difficult to treat, make wound treatment an important clinical, social and economic problem [1].

Wound healing is a dynamic mechanism involving continuous cell–cell and cell–matrix interactions in a series of overlapping phases; hemostasis (blood coagulating cascade), inflammatory, proliferative and finally the remodeling [2,3]. This mechanism is regulated by several mediators and many cell types, including platelets, inflammatory cells, fibroblasts, keratinocytes, also cytokines, growth factors and matrix metalloproteinases (MMP) [4]. Alterations in any of the steps of the normal wound-healing process, which is not a linear process, can cause delay or inability in repair [5]. Therefore, the removal of factors (i.e., malnutrition, infection) that may adversely affect the normal wound healing period, as well as the use of substances capable of accelerating healing internally or externally is needed. Despite the allopathic hegemony and superiority of synthetic compounds for local and systemic use in wound repair, there is a growing interest in natural treatment options that support the wound healing process [6,7,8]. In this context, plants are regarded as a good source for various phytoconstituents, and these are assimilated more easily, as compared to synthetic drugs, by both humans and animals. Traditional treatment alternatives, especially plant-based compounds are recognized as easily accessible treatment options that eliminate the disruptions associated with modern treatment methods [9,10]. Within this context, World Health Organization (WHO) recommends and encourages the use of plant-based treatments, which have been known as safe and effective [11]. Herbal products are also regulated by the European Parliament that established a special category of “traditional herbal medicinal products” for validation of safety and quality of the products by different assays [12].

Turkey is one of the important centers of diversity for the genus *Thymus* (Lamiaceae). This genus is represented by 39 species and 59 taxa in Turkey, and the ratio of endemism in the genus is 53%. Ethnomedicinally, *Thymus* species, are orally used in folk medicine for the treatment of various complaints such as gastrointestinal disorders, cough, bronchitis or pertussis. Also, the extracts obtained from the *Thymus* species are used for the treatment of laryngitis and tonsillitis in gargle form, as well as topical applications for wounds and oral cavity disorders to provide hygiene [13]. Furthermore, some important biological properties such as carminative, expectorant, antitussive, antibacterial and antioxidant activities of some plants belonging this genus have been scientifically confirmed [14,15].

*Thymus sipyleus* Boiss. subsp. *rosulans* (Borbas) *Jalas* (TS) is an endemic species which widely grows in Turkey and is locally known as “kekik otu”. TS is often used in Turkish traditional medicine to treat hemorrhoid, skin wounds, diabetes, arteriosclerosis and gastrointestinal disorders [16]. In particular, the use of this plant for wound healing is based on merely traditional knowledge and to the best of our knowledge, there are no reports in the literature regarding the wound healing properties of TS extracts prepared with different solvent systems. It is known that the type and amount of bioactive compounds extracted from plant materials may vary depending on the polarity of solvent used for extraction [17,18,19]. Accordingly, in this study we used solvents (water, 96% ethanol and *n*-hexane) with different polarities to separate out bioactive compounds with respect to difference in polarity. In this manner, we aimed to comparatively evaluate the effects of various solvent extracts of TS on different stages of the healing process by in vitro test systems, as well as to confirm the traditional use of this plant.

## 2. Results and Discussion

In developing innovative approaches to wound healing, plant-based traditional therapies are considered as attractive options. These therapies offer new opportunities to successfully treat skin lesions, facilitating access to the health care, overcoming the problems associated with modern therapies such as high treatment costs, long manufacturing times, and increased bacterial resistance.

With this perspective in mind, the wound healing potency of TS was investigated with methods including the in vitro evaluation of plant-based medicines in relation to the effects on components involved in the wound healing process. These methods comprise the wound healing effects of different extracts, as well as different biological activities such as antioxidant, antibacterial and anti-inflammatory properties.

### 2.1. Antioxidant Activities of TS Extracts

One of the main factors that play an essential role in wound healing is the regulation of inflammation and oxidation. The excessive free radical production in response to cutaneous damage may complicate the healing process by destroying proteins, lipids and extracellular matrix (ECM) elements [20]. In the present study, we demonstrated the antioxidant status of TS extract by in vitro bioanalytic methodologies. Antioxidant activities of TS extracts were based on free radical scavenging capability measured by cell-free biochemical testing, in addition to cellular antioxidant activity measured by protection against hydrogen peroxide (H_2_O_2_)-induced damage.

2,2-diphenyl-1 picrylhydrazyl (DPPH) free radical scavenging assay has been widely applied to determine the capacity of plant extracts to donate hydrogen or to scavenge free radicals. The extract concentration required to decrease the initial DPPH concentration by 50% (EC_50_) is calculated to evaluate the radical scavenging activity [21]. The EC_50_ values of DPPH radical scavenging activities of TS extracts are shown in Table 1. All extracts from TS exhibited varying degrees of scavenging activity, except Soxhlet *n*-hexane (SN), Soxhlet ethanol/*n*-hexane (SEN), maceration *n*-hexane (MN) and maceration ethanol/*n*-hexane (MEN) extracts. The percentage of DPPH radical scavenging activities of these extracts were ranged from 74.32% to 93.02%, at the highest (200 µg/mL) concentration. Decoction (D) and infusion (I) extracts exhibited the highest activity against DPPH free radical with EC_50_ values of 43.39 ± 1.02 µg/mL and 87.38 ± 1.73 µg/mL, respectively. Also, the DPPH radical scavenging percentages of I (90.5%) and D (93.02%) extracts were found to be comparable with ascorbic acid (95.79%), at 200 µg/mL concentration. 

It is thought that the free radical scavenging activities of plant extracts that occurs in varying degrees, depending on their antioxidant properties, may depend on the presence of polyphenol compounds, particularly phenols. In fact, it is known that phenolic compounds are molecules with many biological properties, including antioxidant activity [22,23]. The amount of total phenolic content in TS extracts are presented in Table 1. Similar to DPPH radical scavenging activity, total phenolic contents of *n*-hexane extracts (SN, SEN, MN, MEN) were too low to be detected. The amount of total phenolics in the other TS extracts were calculated at the range of 78.15 ± 1.17 − 147.64 ± 3.8 mg GAE/g. In accordance with the results of DPPH radical scavenging assay, D and I extracts had the highest amount of phenolic compound (147.64 ± 3.8 mg GAE/g and 118.5 ± 2.3 mg GAE/g, respectively), among all TS extracts. 

Previous studies have reported that TS extracts have strong antioxidant activity, and this effect may be related to the presence of phenolic compounds in extracts such as monoterpenes (thymol, carvacrol, etc.) [24]. In our study, no significant correlation (R^2^ = −0.656) was found between DPPH free radical scavenging activity and the amount of phenolic compound. The fact that total phenolic content does not include all antioxidants in the extracts may explain the unclear relationship between antioxidant capacity and total phenolic content [25]. It is thought that the high antioxidant capacity of TS is not only dependent on the phenolic content of extracts, as well as the other non-phenolic phytoconstituents. On the other hand, antioxidant activities of plant extracts are strongly dependent on the solvent used for extraction, because of the different antioxidant activities of active compounds having different polarities [26]. It has been suggested that antioxidant activities of more-polar solvent extracts were relatively higher than non-polar ones [27,28]. Our results are consistent with this argument, as evidenced by the fact that the most polar extracts (I and D extracts) of TS exhibit the highest antioxidant activity and contain the highest total phenols, due to the high solubility in polar solvents. This situation may be explained by reasoning that the phenolic compounds in the aqueous extracts have an ideal structure for decomposing free radicals since they have many hydroxyl groups acting as hydrogen donors, turning them into powerful antioxidant agents.

**Figure 1:**
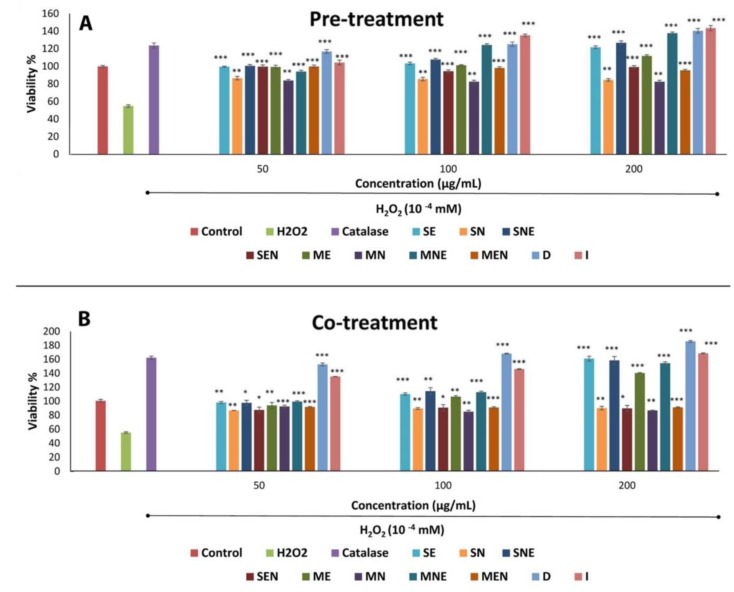
Effect of TS extracts on H_2_O_2_-induced damage in fibroblasts after pre-treatment (**A**) and co-treatment (**B**) protocols. Data were presented as mean ± SEM (n = 3) and compared with H_2_O_2_ treated group, * *p* < 0.05, ** *p* < 0.01, *** *p* < 0.001. SE—Soxhlet ethanol, SN—Soxhlet *n*-hexane, SNE—Soxhlet n-hexane/ethanol, SEN—Soxhlet ethanol/*n*-hexane, ME—Maceration ethanol, MN—Maceration *n*-hexane, MNE—Maceration *n*-hexane/ethanol, MEN—Maceration ethanol/*n*-hexane, D—Decoction, I—Infusion.

Oxidative stress has a critical role in the progression of many disorders including wounds. Reactive oxygen species (ROS), which is an important component of oxidative stress, may not only act as signaling molecules regulating various biological processes, but may also act as toxic molecules [29]. ROS is produced in low amounts at the wound site, as a defense mechanism against invasive bacteria. However, excessive release of ROS may cause cellular damage and may lead to impaired wound repair [23]. Some reports have demonstrated that ROS induces apoptotic changes that may cause cell death and may affect collagen expression in fibroblasts. Therefore, the presence of antioxidant agents is thought to be an important factor in the successful completion of wound repair [23,30]. In addition, H_2_O_2_-induced oxidative stress has been reported as an alternative to study the antioxidant activities of plant extracts in cells [31]. Previous studies on different *Thymus* species have shown that this plant imparts antioxidant effects that play a crucial role in healing process by protecting cells from oxidative damage caused by free radicals [32,33,34,35]. In the present study, the protection degrees of TS extracts against oxidative stress were differed in H_2_O_2_-treated cells, both in the co-treatment and pre-treatment protocols, and this may explain the possible mechanism of antioxidant effect. TS extracts showed complete protection against H_2_O_2_ damage on fibroblasts at all concentrations, both in co-treatment and pre-treatment protocols (Figure 1). In the co-treatment protocol, it was observed that the cell viability increased by 1.54 to 3.38 fold when compared to only H_2_O_2_ treated group. The observed protective effect on the fibroblasts could be due to the direct interaction of the extracts and hydrogen peroxide, as a result of the antioxidant activity, which was also evidenced in the DPPH free radical scavenging assay. D and I extracts exhibited the highest protection in a concentration-dependent manner against H_2_O_2_-induced damage (152.28% to 185.22% and 134.88% to 168.02%, respectively), and this activity was comparable with the positive control. On the other hand, pre-treatment of fibroblasts with TS extracts increased the cell viability by 1.5 to 2.61 fold when compared to only H_2_O_2_ treated cells. In this protocol, although all extracts of TS significantly increased the cell viability, D extract (140.47%), I extract (143.58%), and maceration *n*-hexane/ethanol (MNE) (137.73%) extract showed catalase-like activity especially at 200 µg/mL concentration. We observed that most of the TS extracts were more active at higher concentrations in cell protection, as defined by cell viability values in both co-treatment and pre-treatment protocols. In accordance with the results of DPPH radical scavenging assay, *n*-hexane extracts showed the lowest cytoprotective effect against H_2_O_2_ induced oxidative stress in both treatment protocols. Considering that the *n*-hexane extracts contain the lowest amount of phenolic compound, the cytoprotective effect is thought to be related to the phenolic contents of the extracts.

### 2.2. Antibacterial Activities of TS Extracts

It is well known that many factors such as pathogen microorganisms, that interfere with the different phases of the healing process, can delay wound healing and also lead to impaired repair [36]. Bacterial contamination can elongate the inflammatory phase by causing prolonged production of pro-inflammatory cytokines [36,37]. If this condition persists and bacterial clearance is not achieved, the wound becomes chronic and fails to heal. Therefore, the elimination of bacterial contamination in the wound microenvironment is essential for optimal wound repair.

In the present study, antibacterial activities of TS extracts were evaluated on the most common bacteria isolated from infected skin wounds. As shown in Table 2, the extracts obtained from TS exhibited antimicrobial activity against the majority of the tested strains with minimum inhibitory concentration (MIC) values ranging from 0.125 to 8 mg/mL. D extract showed varying degrees of antibacterial activity against all tested bacteria, while these bacterial strains were found to be resistant against SEN, MN and MEN extracts. *Staphylococcus aureus*, *Staphylococcus epidermidis* and *Bacillus subtilis* were found as the most sensitive bacteria to TS extracts. In this study, Gram-negative bacteria (*Escherichia coli*, *Klebsiella pneumoniae* and *Pseudomonas aeruginosa*) were found to be more resistant to most of TS extracts than Gram-positive bacteria.

Gram-negative bacteria have an outer phospholipids membrane which acts as a strong permeability barrier against hydrophobic molecules, in addition to their cytoplasmic membranes [38,39]. Previous studies have reported that this membrane prevents the penetration of hydrophobic molecules to the Gram-negative bacteria cell wall [40,41]. In this context, it was thought that the resistance of Gram-negative bacteria to TS extracts may be related to the presence of protective cell membrane, which inhibits the passage of hydrophobic molecules, such as phenolics, known to have antibacterial activity.

Various classifications, based on MIC values, may be used to evaluate the antibacterial activities of plant extracts. According to Duarte et al. [42] plant extracts that present MIC values below 2 mg/mL, can be considered as having potential antimicrobial activity. Additionally, Fabry et al. [43] have reported that the plant extracts should have MIC values up to 8 mg/mL, to be considered as therapeutically active. According to these classifications, it can be concluded that all TS extracts, except SEN, MN and MEN, have moderate antibacterial activity against different types of bacteria that can cause wound infections. These results revealed that most of the TS extracts might be useful in the wound healing process as an alternative antibacterial agent.

The bioactive compounds in the extracts may be responsible for the antibacterial activities of the extracts [44]. Previous studies have suggested that rosmarinic acid, which is a phenolic compound found in *Thymus* species, possess bactericidal activity against *B. subtilis, S. aureus, E.coli, P. aeruginosa* and *S. epidermidis* [45,46]. Additionally, it has been informed that thymol and carvacrol can limit the growth of bacteria as a result of their ability to permeabilize, depolarize, and disruption of the bacterial cytoplasmic membrane [47]. Therefore, it is thought that the antibacterial activities of our extracts may be related to the presence of rosmarinic acid, carvacrol and thymol, which were previously reported to be isolated from TS [15,48].

### 2.3. Effects of TS Extracts on Cell Proliferation

Fibroblast proliferation is involved in the restoration of structure and function during the tissue formation phase in the healing process. Fibroblasts secrete collagen and affect the remodeling of granulation tissue into mature dermis [31]. Since the fibroblasts are the major targets in therapeutic drug design, bioactive compounds that can stimulate the proliferation of fibroblasts may be able to stimulate the healing process, as in the case of our present study [49]. MTT (3-[4-dimethylthiazol-2-yl]-2,5-diphenyl tetrazolium bromide) assay, applied in this study, functions in multiple ways, and we set out to determine the active extracts and their optimal concentrations that can influence the metabolic activity of cells as well as measure the cell proliferation rate [50].

**Figure 2:**
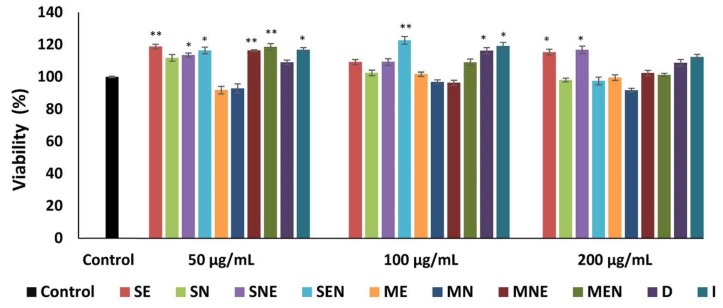
Proliferation of fibroblasts (%) treated with different concentrations (50, 100, 200 µg/mL) of TS extracts by MTT assay. Untreated fibroblasts were used as control. Data were presented as mean ± SEM (n = 3) and compared with the control group, * *p* < 0.05, ** *p* < 0.01. SE—Soxhlet ethanol, SN—Soxhlet *n*-hexane, SNE—Soxhlet *n*-hexane/ethanol, SEN—Soxhlet ethanol/*n*-hexane, ME—Maceration ethanol, MN—Maceration *n*-hexane, MNE—Maceration *n*-hexane/ethanol, MEN—Maceration ethanol/*n*-hexane, D—Decoction, I—Infusion.

We determined more than 90% viable cells after treatment with TS extracts at all applied concentrations (Figure 2). Our findings indicated that none of the extracts exhibited cytotoxic activity on mouse fibroblasts even at high concentrations of 200 μg/mL since it did not affect the cellular activity of fibroblasts. The percentages of cell viability ranged from 91.86% to 122.69% in groups treated with different concentrations of TS extracts. All extracts of TS, except SN, MN and maceration-ethanol (ME) caused a significant increase (*p* < 0.05) in fibroblast proliferation at least one tested concentration. The activities of these extracts, which significantly increased fibroblast proliferation, were close to that of fibroblast growth factor (FGF) (123.92%). In this study, we observed that TS extracts exhibited more pronounced proliferative activity at 50 and 100 µg/mL concentrations than at the concentration of 200 µg/mL.

Etnopharmalogical studies have indicated that extracts obtained from some *Thymus* spp. have a wound healing potential, due to their phytochemical constituents, such as polyphenolic compounds of plants, which have the ability to promote fibroblast proliferation during the wound healing process [51,52]. However, it is suggested that the effects of flavonoids on fibroblast proliferation depend on their structure and concentration [53]. In addition to previous studies, the results of this study confirm that flavonoids stimulate fibroblast proliferation, especially at lower concentrations. In this case, it is thought that phenolic compounds such as flavonoids in the extracts, may play an important modulatory effect on fibroblast metabolism.

Selection of extraction solvent is considered as an important parameter to achieve phenolic compounds from plant extracts at effective concentrations. Ethanol has been demonstrated as an effective solvent in the extraction of phenolic compounds in many studies [54]. Consistent with this finding, in this study, ethanolic extracts showed some affinity to extract bioactive compounds from the plant even after the use of *n*-hexane. This may be due to the high extractability of some active compounds in ethanol.

### 2.4. Scratch Wound Healing of Fibroblasts in the Presence of TS Extracts

**Figure 3:**
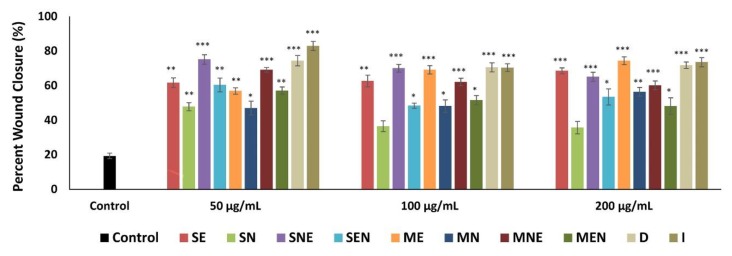
Wound closure percentage of fibroblasts after treatment with different concentrations (50, 100, 200 µg/mL) of TS extracts for 72 h. Untreated fibroblasts were used as control. Data were presented as mean ± SEM (n = 3) and compared with the control group, * *p* < 0.05, ** *p* < 0.01, *** *p* < 0.001. SE—Soxhlet ethanol, SN—Soxhlet *n*-hexane, SNE—Soxhlet *n*-hexane/ethanol, SEN—Soxhlet ethanol/*n*-hexane, ME—Maceration ethanol, MN—Maceration *n*-hexane, MNE—Maceration *n*-hexane/ethanol, MEN—Maceration ethanol/*n*-hexane, D—Decoction, I—Infusion.

**Figure 4:**
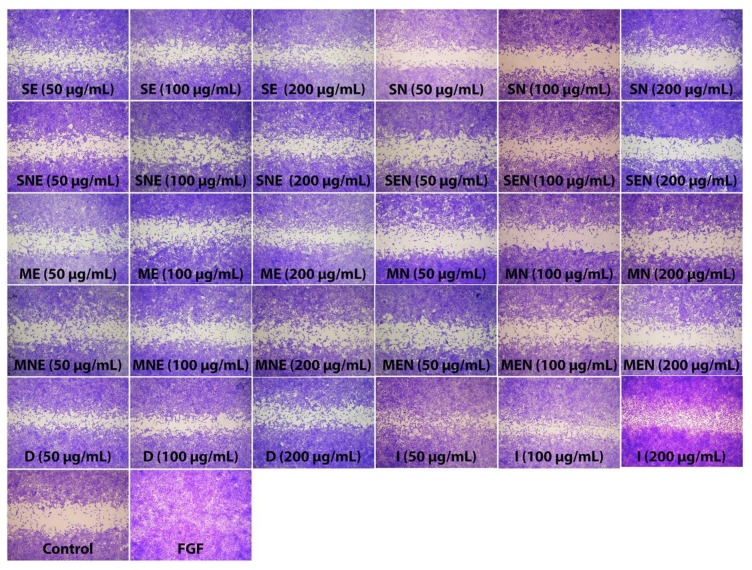
Representative images of each treatment group after 72h. Untreated fibroblasts were used as control. Fibroblast growth factor (FGF) (25 ng/mL) was used as positive control. SE—Soxhlet ethanol, SN—Soxhlet *n*-hexane, SNE—Soxhlet *n*-hexane/ethanol, SEN—Soxhlet ethanol/*n*-hexane, ME—Maceration ethanol, MN—Maceration *n*-hexane, MNE—Maceration *n*-hexane/ethanol, MEN—Maceration ethanol/*n*-hexane, D—Decoction, I—Infusion.

During the wound healing process, skin cells such as fibroblasts migrate into the wound site and proliferate in order to restore skin integrity and generate new granulation tissue [55]. Thus, proper proliferation and migration of fibroblasts are necessary factors to the formation of granulation tissue as the basis of new dermis. Previous studies have demonstrated that plant-based natural compounds that can stimulate migration of fibroblasts promote wound healing by helping to develop an optimized therapeutic protocol [56]. In this context, in this study, the effects of TS extracts on fibroblast migration were assessed by using scratch assay, which is a useful method to mimic the migration of cells during wound healing in vivo. The effect of TS extracts on fibroblast migration were expressed as a percentage of wound closure. The rate of wound closure was found to be approximately 100% in the group treated with positive control (FGF). All extracts of TS, except SN, significantly increased (*p* < 0.05) the natural migration rate of fibroblasts into the wounded area, at all applied concentrations. As shown in Figure 3 and Figure 4, the percentage closure of the scratched area was 19.4% in the control cells, while the closure percentage varied from 35.74% to 82.96% in the groups treated with TS extracts. Especially D and I extracts exhibited the most promising effects in the scratch assay with approximately 3.6 and 4.2 fold higher wound closure rates compared to the control group.

Previous studies have reported that wound closure is a mechanism involves the combination of proliferation and migration of fibroblasts [50]. Our results revealed that TS extracts can promote wound closure and granulation tissue formation by inducing cell migration, which is a critical stage in the proliferative phase, as well as fibroblast proliferation. Considering that fibroblasts are important cells for wound contraction and the production of ECM [57], it is thought that this increased mitogenic effect with the treatment of TS extracts may contribute to the wound healing process.

### 2.5. Effects of TS Extracts on Hydroxyproline Level

Hydroxyproline is an amino acid essential for collagen which is the predominant extracellular protein in the granulation tissue of wounds. For this reason, the level of hydroxyproline has been used as a biomarker for the determination of the content of collagen [58]. Collagen plays a key role in hemostasis and also is vital for re-epithelialization of cellular-matrix and intercellular interactions at a later phase of wound healing, thereby strengthening and integrating the wound matrix. Since the increase in tensile strength and epithelization may be a result of increased hydroxyproline content in the wound tissue [59].

**Figure 5:**
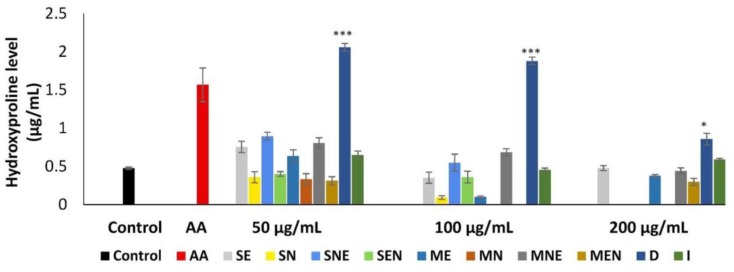
Effects of TS extracts on hydroxyproline content. Untreated fibroblasts were used as control. Ascorbic acid (AA) (25 µg/mL) was used as positive control. Data were presented as mean ± SEM (n = 3) and compared with the control group, * *p* < 0.05, *** *p* < 0.001. SE—Soxhlet ethanol, SN—Soxhlet *n*-hexane, SNE—Soxhlet *n*-hexane/ethanol, SEN—Soxhlet ethanol/*n*-hexane, ME—Maceration ethanol, MN—Maceration *n*-hexane, MNE—Maceration *n*-hexane/ethanol, MEN—Maceration ethanol/*n*-hexane, D—Decoction, I—Infusion.

Changes in hydroxyproline level after treatment with TS extracts at different concentrations were shown in Figure 5. Although the extracts of TS, except SN, MN, SEN, MEN, increased hydroxyproline synthesis at least one tested concentration, this increase was found significant (p < 0.05) only in the D extract at all concentrations. The synthesis of hydroxyproline was stimulated approximately 1.8 to 4.3-fold by D extract when compared to untreated fibroblasts. It was observed that TS extracts showed more pronounced activity on hydroxyproline synthesis at a concentration of 50 µg/mL.

It has been suggested that flavonoids display a high affinity for collagen and that they could increase the amount of collagen required for the formation of new wound matrix by inhibiting MMP, consequently accelerating the healing process [60]. Additionally, the treatment of fibroblasts with flavonoids may result in increase of measurable extracellular collagen. In this study, we observed that more-polar solvent extracts of TS caused the greater increase in the concentration of hydroxyproline in fibroblast cultures than non-polar extracts. It can be assumed that phenolic compounds, such as flavonoids, determined in higher amounts in polar extracts, may be responsible for increasing collagen synthesis as indicated by elevated hydroxyproline content.

### 2.6. Anti-Inflammatory Activities of TS Extracts

Wound healing occurs as a cellular response to injury and during the early phase of wound repair macrophages that play a central role in tissue damage are involved in the maintenance of tissue hemostasis and initiation of inflammatory events. These cells can produce several inflammatory mediators, among them, nitric oxide (NO) is crucial for the development of subsequent events, especially the resolution of inflammation [61].

NO, which has cytostatic, chemotactic, and cytotoxic activity against various bacteria and tumor cells, plays an important role in wound healing including the proliferation and differentiation of various type of cells, modulation of collagen deposition and wound contraction [62]. However, excessive NO production acts as a reactive radical, which damages the function of normal cell by attacking normal tissue surrounding the wound site. Thus, the regulation of reactive radical production is an important factor in recruitment of fibroblast, which is attracted into the site to initiate the proliferative phase of healing process [63]. Also, it has been reported that NO directly affect the tumor necrosis factor-alpha (TNF-α) expression, another important mediator in the inflammatory response [62]. Pro-inflammatory cytokines including interleukins (ILs) and TNF-α play a key role in wound repair by modulating many processes in the wound site, such as stimulation and proliferation of keratinocytes and fibroblasts, synthesis of ECM proteins and regulation of immune response [64]. While low concentrations of TNF-α promotes the wound healing process by stimulating inflammation, its well known that TNF-α plays a destructive role on the repair in higher concentrations. Therefore, the inhibition of pro-inflammatory mediators such as TNF-α may be an effective therapeutic approach to regulating the wound healing process [65].

**Figure 6:**
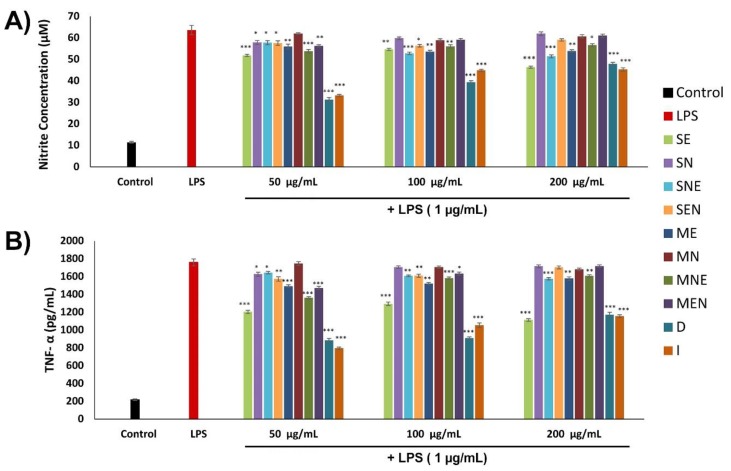
Effects of TS extracts on lipopolysaccharide (LPS)-induced nitric oxide (NO) (**A**) and tumor necrosis factor-alpha (TNF-α) (**B**) production in macrophage cells. Untreated fibroblasts were used as control. Data were presented as mean ± SEM (n = 3) and compared with only LPS treated group, * *p* < 0.05, ** *p* < 0.01, *** *p* < 0.001. SE—Soxhlet ethanol, SN—Soxhlet n-hexane, SNE—Soxhlet n-hexane/ethanol, SEN—Soxhlet ethanol/n-hexane, ME—Maceration ethanol, MN—Maceration n-hexane, MNE—Maceration n-hexane/ethanol, MEN—Maceration ethanol/n-hexane, D—Decoction, I—Infusion.

In this study, the possible role of TS extracts on the inflammatory phase was examined by measuring the inhibitory effects on the release of NO and TNF-α. TNF-α and NO production were investigated in macrophage cells stimulated with lipopolysaccharide (LPS), in the presence or absence of TS extracts for 24 h. The effects of TS extracts on the production of NO and TNF-α in macrophages are exhibited in Figure 6. In order to evaluate the effects of TS extracts on NO production, the accumulation of nitrite (NO_2_) were measured in the culture supernatant. Stimulation of macrophages with LPS significantly increased the level of NO_2_ (approximately 5.5-fold) compared to control. Our results revealed that this stimulation was inhibited by 2.61% to 50.86% in the presence of TS extracts. D and I extracts exhibited the highest activity with percentage inhibition of 50.86% and 47.79% respectively, at 50 µg/mL concentration. The down-regulation of NO production by these extracts possibly protects macrophages from NO damage. Similarly, it was observed that stimulation with LPS significantly enhanced the secretion of TNF-α from macrophages (approximately 8-fold) as compared to control. SB203580 (positive control) reduces TNF-α production from LPS-stimulated macrophages by approximately 75%. Treatment of cells with TS extracts suppressed TNF-α production in macrophages after LPS induction by 0.69% to 54.79%. The highest activity was recorded in D and I extracts at 50 µg/mL concentration, with percentage inhibition of 49.76% and 54.79% respectively.

Our results demonstrated that TS extracts inhibit the inflammatory responses in LPS-stimulated macrophages, by suppressing overproduction of NO and TNF-α and thus may have potential anti-inflammatory activity. *Thymus* species have been reported as sources of different phenolic compounds such as caffeic acid and its derivatives (rosmarinic acid, salvianolic acid etc.) [66]. Previous studies have shown that these phyto-compounds might decrease the production of pro-inflammatory cytokines by suppressing nuclear factor kappaB (NF-κB) activation [67,68,69]. It is thought that the possible responsible mechanism for down-regulation of inflammatory mediators may be the suppression of NF-κB pathway at different levels, by the active components contained in the extracts. On the other hand, another possible mechanism for reduction of TNF-α expression may be the antioxidant activities of the extracts [70,71,72].

### 2.7. HPLC Analysis of the Infusion (I) and Decoction (D) Extracts for Luteolin-7-O-glucoside Content

**Figure 7:**
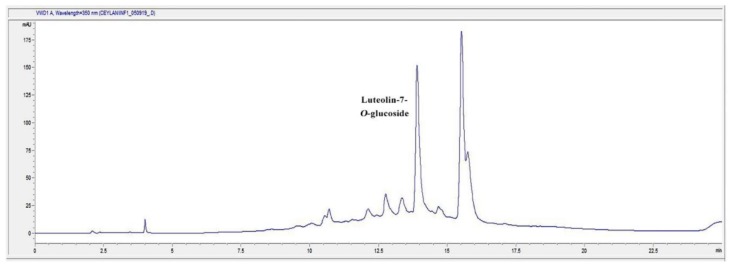
The HPLC Chromatogram of the infusion (I) extract of TS.

**Figure 8:**
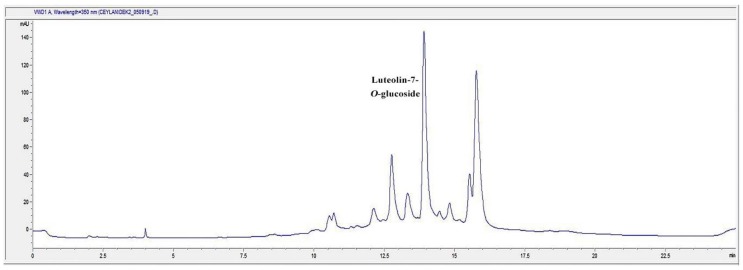
The HPLC Chromatogram of the decoction (D) extract of TS.

**Figure 9:**
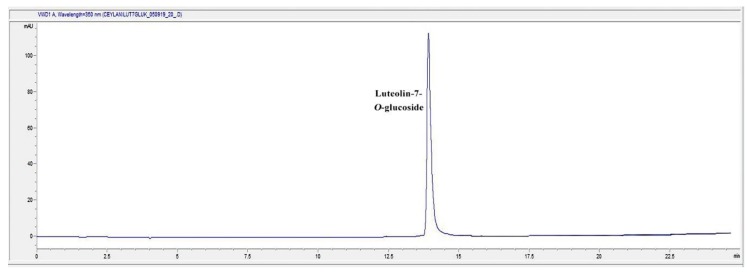
The HPLC Chromatogram of luteolin-7-*O*-glucoside (Retention time: 13.9 min).

In the present study, D and I extracts of TS were found to be the most effective extracts on different phases of wound healing process. Accordingly, we determined the phytochemical characterization of the most active extracts. The general chromatogram-fingerprint of the I and D extracts of the plant is shown in Figure 7 and Figure 8. Moreover, the first single peak was analyzed in detail, since the second huge peak might be the mix of compounds due to its size. When the retention time of the single peak was compared to the inhouse reference compounds, results revealed that the single peak was luteolin-7-*O*-glucoside (Figure 9).

Qualitative analysis was followed by quantitative analysis of luteolin-7-*O*-glucoside in the aqueous extracts of TS demonstrated that in both I and D extracts contain luteolin-7-*O*-glucoside at a significant rate (Table 3).

We analyzed the first single major peak of the chromatogram further by comparing it to the reference flavonoid compounds. The result showed that the single compound might strongly be luteolin-7-*O*-glucoside, which is one of the flavonoid glycosides. This study showed that luteolin-7-O-glucoside was found in both I and D extracts at a remarkable rate. A previous study has shown that luteolin-7-*O*-glucoside has strong anti-inflammatory activity [73]. In the in silico study, it was reported that the transcription factors Src (in the nuclear factor (NF)-κB pathway), MAPK (in the activator protein (AP)-1 pathway), and SOCS3 (in the signal transducer and activator of transcription 3 pathway) are luteolin derivatives’ major target. In silico, in vitro, in vivo studies and a clinical trial with a formulation containing luteolin has shown that luteolin has great therapeutic effect against inflammation-associated disturbances. In addition, the effects of luteolin ointment on the key steps of wound healing, such as cell proliferation, cell migration and collagen deposition, have been proven in previous reports [74,75].

## 3. Materials and Methods

### 3.1. Plant Material

TS was collected in June 2013 from Sivas: Ulaş-Kovalı, Ziyarettepe (1300 m), Turkey. It was deposited at Cumhuriyet University Herbarium, Faculty of Science, Department of Biology, Sivas-Turkey, with voucher specimen number CUFH-AA 5018.

### 3.2. Preparation of Extracts

The aerial parts of TS were dried at room temperature and ground into powder form. The extracts were prepared by using different extraction techniques; Soxhlet, maceration, infusion and decoction methods. In Soxhlet and maceration methods, two different solvents (96% ethanol and *n*-hexane) with different polarities were used in succession, in order to isolate different bioactive compounds from the plant material. Aqueous extracts were prepared by using decoction and infusion techniques.

Soxhlet and maceration extraction methods were performed according to Schmidt et al. [76] with slight modifications. In the first stage of Soxhlet extraction, plant material (25 g) was first extracted in the Soxhlet extractor (İldam, Ankara, Turkey) with *n*-hexane (250 mL) for 12h; then the same material was re-extracted with ethanol. The second stage of Soxhlet extraction was carried out with 25 g of air-dried plant material using ethanol followed by *n*-hexane, under the same conditions described above. Similarly, plant sample (25 g) was successively macerated with *n*-hexane and ethanol at room temperature, for 10 consecutive days. For the decoction method, powdered plant material (25 g) was suspended in deionized water (500 mL) and boiled at 100 °C for 5 min [77]. The infusion was performed according to the method described by Albayrak et al. [78]. Powdered plant material (25 g) was added to boiled deionized water (500 mL), maintained for 5 min and then allowed to stand at room temperature for 10 min.

All extracts were passed through Whatman filter paper No. 1 (Whatman, Maidstone, UK) and the solvents were removed under reduced pressure at 40 °C, using a rotary vacuum evaporator (Laborota 4000, Heidolph Instruments GmbH & Co, Schwabach, Germany). The extracts were lyophilized with a freeze-dryer (Christ Alpha 2–4 LD plus, Martin Christ, Osterode am Harz, Germany) and the plant material was stored at –20 °C until analysis.

### 3.3. DPPH Radical Scavenging Assay

DPPH free radical scavenging activities of the extracts were determined according to the procedure described by Yang et al. [79]. DPPH solution (50 µL) was mixed with methanolic solutions of TS extracts (200 µL) at varying concentrations ranging from 25 to 200 µg/mL. After the incubation period (30 min) the absorbance (Abs) was measured using a multi-mode microplate reader (FilterMax F5, Molecular Devices, Sunnyvale, CA, USA) at 517 nm. The EC_50_ value, described as the concentration of the extracts required for 50% scavenging of DPPH radical, was calculated using the graph by plotting inhibition percentage against extract concentration. Ascorbic acid was used as positive control.

### 3.4. Folin-Ciocalteu Assay

Total phenol contents of the extracts were estimated by using Folin–Ciocalteu assay [80]. Methanolic solutions of TS extracts or gallic acid, standard compound, (10 µL) were added to Folin–Ciocalteu reagent (150 µL, 1:4 diluted with water) and incubated at room temperature, during 3 min. Subsequently, sodium carbonate solution (50 µL) was added, and the mixture was allowed to stand in the dark at room temperature for 2 h, after which the absorbance was measured at 725 nm. Total phenol contents of the extracts were expressed as milligram of gallic acid equivalent (GAE) per gram of extracts.

### 3.5. Agar Dilution Assay

In order to determine the antibacterial activities of TS extracts quantitatively, the agar dilution method was performed. The extracts were dissolved at a concentration of 80 mg/mL and were prepared for two-fold step dilution for ten serial dilutions with CAMHB, according to Clinical and Laboratory Standards Institute (CLSI) [81]. Thus, the concentrations of the extracts were prepared between 8 mg/mL and 0.015625 mg/mL. Gentamicin sulphate was used as positive control. Each inoculum (1 mL) was poured into each petri dish, and 9 mL Muller–Hinton agar brought to 45–50 °C was added onto inoculum, then mixed with a circular rotation and allowed to cool at the room temperature. A bacterial suspension with 107 CFU/mL final concentration was prepared and was inserted into the microplate wells. The sterilized replicator with 3-mm pins, which deliver 2 μL, was placed into the microplate to soak the pins and transfer it onto the agar plate. The agars were incubated at 37 °C for 24 h. The MIC values were determined beyond the level no inhibition of growth of test organisms was observed.

### 3.6. Cell Culture and Reagents

For cell culture assays, the stock solutions of extracts were prepared with dimethyl sulfoxide (DMSO) and then diluted with cell culture media for the desired concentrations (50, 100 and 200 µg/mL). The final DMSO concentration in culture media was less than 0.2%, and control groups were treated with the corresponding amount of DMSO. The 3T3-Swiss albino mouse fibroblast cell line (ATCC-CCL-92) was obtained from ATCC (Rockville, MD, USA) and RAW 264.7 macrophage cell line (ATCC TIB-71) was a generous gift from Prof. Hande Sipahi (Faculty of Pharmacy, Yeditepe University). All reagents used in cell culture were purchased from Sigma Chemical Co. (St. Louis, MO, USA). 3T3-Swiss albino mouse fibroblast cells were cultured in Dulbecco’s Modified Eagle’s Medium-F12 (DMEM-F12) and murine RAW 264.7 cells were cultured in DMEM high glucose medium supplemented with 10% fetal bovine serum (FBS) and 1% penicillin/streptomycin, in atmosphere of 5% CO_2_ and 95% humidity at 37 °C. LPS from *Escherichia coli*, SB203580 and FGF were obtained from Sigma Chemical Co. (St. Louis, MO, USA).

### 3.7. Cell Viability Assay

To determine the effects of TS extracts on cell proliferation, cells were seeded at a density of 1 × 10^4^ cells/well and incubated for 24 h at 37 °C and 5% CO_2_ to allow attachment. The medium was replaced with fresh medium supplemented with various concentrations of extracts or positive control (FGF). Following 72 h incubation, the viability of fibroblasts was determined by using a cell MTT cell proliferation kit (Roche, Mannheim, Germany) according to the manufacturer’s instructions. The absorbance (Abs) of each well was measured by using a microplate reader (FilterMax F5, Molecular Devices, USA) at 595 nm. The control cells were considered as 100% viable. The cell viability percentage was calculated by the following equation:

Cell Viability (%) = (Abs_sample_/Abs_control_) × 100 (1)

### 3.8. In Vitro Wound Healing (Scratch) Assay

The role of TS extracts on fibroblast migration were determined by using CytoSelect 24-well wound healing assay kit (Cell Biolabs Inc., San Diego, CA, USA). For this purpose, cells were cultured at a total of 500 µL of cell suspension comprised of 2x10^5^ cells in each well, including wound field inserts. After overnight incubation, the inserts were removed to form free of cells area. The cells were washed with phosphate-buffered saline (PBS) and afterwards treated TS extracts at different concentrations. FGF (25 ng/mL) was used as a positive control. Cells were incubated at 37 °C with 5% CO_2_ for 72 h. After incubation, the medium of the cells was removed, and the cell stain solution was added to each well. Stained cells were incubated (15 min) at room temperature. The microscopic images were taken through the center of the scratched area, at 40× magnification using a phase contrast Olympus BX50 inverted microscope (Olympus America, Inc., NY, USA). Quantification of the percentage of wound healing was determined by the percentage of closure using the following equation:

Percent Closure (%) = (Migrated Cell Surface Area/Total Surface Area) × 100(2)

### 3.9. Nitric Oxide (NO) Assay

As an indicator of NO production, the quantity of NO_2_, a stable NO metabolite, in the culture media was measured in LPS-stimulated RAW 264.7 macrophages. The cells were cultured at a concentration of 2 × 10^5^ cells/well in 24 well plates and incubated for 24 h at 37 °C and 5% CO_2_. Then medium of each well was discarded, and various concentrations of (50, 100 and 200 µg/mL) TS extracts were added to each well and incubated for 1 h. Afterwards, cells were treated additionally with LPS (1 µg/mL) for 24 h. At the end of incubation, all cell culture medium from each well was collected for measurement of NO_2_ concentration. For this purpose, the culture supernatant (100 µL) was mixed with the same amount of Griess reagent (1% sulfanilamide and 0.1% naphthyl ethylenediamine dihydrochloride in 2.5% phosphoric acid) and incubated for 10 min, at room temperature. The absorbance was measured at 530 nm in a microplate reader (FilterMax F5, Molecular Devices, Sunnyvale, CA, USA) and NO_2_ levels were measured from a sodium nitrite (NaNO_2_) standard curve [82].

### 3.10. Tumor Necrosis Factor-Alpha (TNF-α) Assay

The amount of TNF-α release by the LPS stimulated RAW 264.7 macrophage cells were measured by using a Mouse TNF-α ELISA kit (Invitrogen Life Technologies, Carlsbad, CA, USA) according to the manufacturer’s instructions. Briefly, cells were cultured at a concentration of 2×10^5^ cells/well and incubated for 24 h at 37 °C and 5% CO_2_. After discarding the medium, TS extracts were added to each well at various concentrations and incubated for 1 h. Post-incubation, the cells were treated with LPS (1 μg/mL) for 24 h. After the incubation period, the culture supernatant was collected and stored at −80 °C until analyzed. SB203580, a p38 MAPK inhibitor, was used as a positive control. Results were expressed as pg/mL.

### 3.11. Hydroxyproline Assay

To measure the effect of extracts towards the production of collagen, hydroxyproline levels were measured in cell culture medium [83]. For this purpose, fibroblast cells were cultured at a concentration of 1 × 10^3^/well and incubated for 24 h at 37 °C and 5% CO_2_. Then medium of each well was discarded, and the cells were treated with various concentrations of the extracts for 72 h. Subsequently, cultured medium was pipetted carefully and hydrolyzed with HCl (6 N) in an autoclave at 120 °C. The samples (20 µL) were transferred into 96-well plate, chloramine-T solution (50 µL) was added to each sample, and the mixture was kept at room temperature for 20 min. Then, Ehrlich’s solution was added to each sample, and the samples were incubated for 15 min at 65 °C. Afterwards, the absorbance was measured at 550 nm by using microplate reader (FilterMax F5, Molecular Devices, Sunnyvale, CA, USA). Ascorbic acid (25 µg/mL) was used as positive control. Concentration of hydroxyproline content was measured from hydroxyproline standard curve (0 to 10 µg/mL). The results were expressed as µg/mL of hydroxyproline.

### 3.12. Antioxidant Activity of Extracts on 3T3-Swiss Albino Mouse Fibroblast Cells

The protective effects of the extracts against H_2_O_2_-induced oxidative damage were evaluated by the method as described by Annan and Dickson [84]. The cells were cultured at a concentration of 5 × 103/well and incubated for 24 h at 37 °C and 5% CO_2_. Then the medium of each well was discarded, and the various concentrations of extracts were added to each well in two different types of experiment. Firstly, the cells were pre-treated with various concentrations of the extracts for overnight then the cells were treated with 10^−4^ M hydrogen peroxide in medium and incubated for 3 h. In the second treatment regimen, the cells were exposed to various concentrations of extracts and 10^−4^ M hydrogen peroxide at the same time in the medium and incubated for 3 h at 37 °C. Catalase (250 unit/mL), an antioxidant enzyme was used as the positive control. At the end of the incubation period, MTT test was applied to determine the protective effects of extracts against hydrogen peroxide-induced damage in fibroblast cells.

### 3.13. Phytochemical Analysis

In the D and I extracts, which have the highest biological activity, fingerprint and qualitative/ quantitative analysis of luteolin-7-*O*-glucoside were performed using a previously validated method for HPLC-UV. [15]. Analysis was performed on Agilent 1220 Series HPLC system (Agilent Technologies, Santa Clara, CA, USA) equipped with a quaternary pump, an auto-sampler, a column oven, and a UV/VIS detector. Agilent Chemstation software was used for data analysis. The separation was executed on HP LiChrospher 100 5 μ RP8 (250 × 4 mm) column. The mobile phase was a mixture of acetonitrile: deionized water: formic acid (HPLC grade) (80:20:0.1) (solution A) and formic acid 0.1% (*v/v*) in deionized water in (solution B). The composition of the gradient (A:B) was shown in Table 4 for luteolin-7-*O*-glucoside. The detection of UV wavelength was set at 350 nm. The column temperature was arranged to 30 °C. Quantification was performed by measuring at 350 nm for luteolin-7-*O*-glucoside. The duration between runs was 2 min. The injection volume was 10 μL.

### 3.14. Statistical Analysis

Data were expressed as mean ± SEM and analyzed statistically by one-way (ANOVA) followed by Student’s t-test. All results were considered significant at *p* < 0.05 (SPSS 15.0 statistical package, Chicago, IL, USA).

## 4. Conclusions

The present study scientifically supports the traditional claims of TS in wound healing. It was possible to determine the effects of TS extracts on cellular proliferation, migration and collagen synthesis by different assays, in addition to their antimicrobial, antioxidant and anti-inflammatory properties. Notably, the TS extracts did not have any cytotoxic effects on the fibroblast cell line. While the most active extracts were D and I, all TS extracts exhibited some activity associated with wound healing in at least one bioassay, but these effects were sometimes only moderate. This is due to the phytochemical ingredients found in the extracts that are believed to play a major role in promoting wound healing activity. However, further detailed isolation and identification of the phytochemicals are necessary to figure out the bioactive compound(s) accountable for the pharmacological activity.

## Figures and Tables

**Table 1:** DPPH free radical scavenging activities of *Thymus sipyleus* Boiss. subsp. *rosulans* (Borbas) *Jalas* (TS) extracts expressed as EC_50_ values and total amount of phenolic compounds.

Extracts	DPPH Scavenging Activity	Total Phenolic Content
	EC_50_ (μg/mL)	mg GAE/g Extract
SE	121.63 ± 1.67	113.9 ± 2.17
SN	N.D. ^a^	N.D.
SNE	100.21 ± 1.19	78.15 ± 1.17
SEN	N.D.	N.D.
ME	104.91 ± 1.04	109 ± 2.44
MN	N.D.	N.D.
MNE	95.19 ± 1.62	90.75 ± 1.29
MEN	N.D.	N.D.
D	43.5 ± 1.02	147.6 ± 3.8
I	87.38 ± 1.73	118.5 ± 2.3
Ascorbic acid ^b^	27.63 ± 1.12	−

^a^ N.D. is not determined. ^b^ Ascorbic acid was used as positive control for DPPH scavenging assay. Results are presented as mean ± SEM (n = 3). SE—Soxhlet ethanol, SN—Soxhlet *n*-hexane, SNE—Soxhlet *n*-hexane/ethanol, SEN—Soxhlet ethanol/*n*-hexane, ME—Maceration ethanol, MN—Maceration *n*-hexane, MNE—Maceration *n*-hexane/ethanol, MEN—Maceration ethanol/*n*-hexane, D—Decoction, I—Infusion.

**Table 2:** Antibacterial activities of TS extracts expressed as minimum inhibitory concentrations (MICs).

Extracts	Minimum Inhibitory Concentrations (MICs) (mg/ mL)						
	*Escherichia coli*	*Klebsiella pneumonia*	*Pseudomonas aeruginosa*	*Bacillus subtilis*	*Bacillus cereus*	*Staphylococcus aureus*	*Staphylococcus epidermidis*
SE	2	2	N.I. ^a^	2	8	0.5	1
SN	1	N.I.	0.125	4	N.I.	2	0.25
SNE	N.I.	N.I.	8	2	4	2	2
SEN	N.I.	N.I.	N.I.	N.I.	N.I.	N.I.	N.I.
ME	N.I.	8	N.I.	2	N.I.	1	1
MN	N.I.	N.I.	N.I.	N.I.	N.I.	N.I.	N.I.
MNE	8	N.I.	N.I.	2	N.I.	8	8
MEN	N.I.	N.I.	N.I.	N.I.	N.I.	N.I.	N.I.
D	8	8	4	1	8	2	1
I	N.I.	N.I.	4	4	8	2	1
Gentamicin	0.00025	0.00025	0.00025	0.0005	0.0008	0.0005	0.0005

^a^ N.I. is not inhibition. SE—Soxhlet ethanol, SN—Soxhlet *n*-hexane, SNE—Soxhlet *n*-hexane/ethanol, SEN—Soxhlet ethanol/*n*-hexane, ME—Maceration ethanol, MN—Maceration n-hexane, MNE—Maceration *n*-hexane/ethanol, MEN—Maceration ethanol/*n*-hexane, D—Decoction, I—Infusion.

**Table 3:** The composition of the gradient (A:B) for luteolin-7-*O*-glucoside.

Extract	The Amount of Luteolin-7-*O*-glucoside (mg/100 mg Extract)
Infusion (I)	0.4050 ± 0.0013
Decoction (D)	0.4599 ± 0.0018

**Table 4:** The composition of the gradient (A:B) for luteolin-7-*O*-glucoside.

Time (min)	Solution A	Solution B	Flow rate (mL/min)
0–10	5%→35%	95%→65%	0.8
10–15	35%→40%	65%→60%	0.6
15–18	40%	60%	0.8
18–20	40%→42%	60%→58%	0.8
20–25	42%→100%	58%→0%	0.8
